# Simulations on Monitoring and Evaluation of Plasticity-Driven Material Damage Based on Second Harmonic of S_0_ Mode Lamb Waves in Metallic Plates

**DOI:** 10.3390/ma10070827

**Published:** 2017-07-19

**Authors:** Xiaoqiang Sun, Xuyang Liu, Yaolu Liu, Ning Hu, Youxuan Zhao, Xiangyan Ding, Shiwei Qin, Jianyu Zhang, Jun Zhang, Feng Liu, Shaoyun Fu

**Affiliations:** 1 College of Aerospace Engineering, Chongqing University, Chongqing 400044, China; tiny_strong@sina.com (X.S.); liuyaolu@cqu.edu.cn (Y.L.); youxuan.zhao@cqu.edu.cn (Y.Z.); ddingxiangyan@yeah.net (X.D.); qsw123@163.com (S.Q.); jyzhang@cqu.edu.cn (J.Z.); mejzhang@cqu.edu.cn (J.Z.); syfu@cqu.edu.cn (S.F.); 2 Key Disciplines Lab of Novel Micro-Nano Devices and System and International R&D Center of Micro-Nano Systems and New Materials Technology, Chongqing University, Chongqing 400044, China; 3 Department of Engineering Mechanics, College of Mechanical and Vehicle Engineering, Hunan University, Changsha 410082, China; liufengeaddr@126.com

**Keywords:** lamb waves, material plasticity, second-order nonlinear wave, monitoring, evaluation

## Abstract

In this study, a numerical approach—the discontinuous Meshless Local Petrov-Galerkin-Eshelby Method (MLPGEM)—was adopted to simulate and measure material plasticity in an Al 7075-T651 plate. The plate was modeled in two dimensions by assemblies of small particles that interact with each other through bonding stiffness. The material plasticity of the model loaded to produce different levels of strain is evaluated with the Lamb waves of S_0_ mode. A tone burst at the center frequency of 200 kHz was used as excitation. Second-order nonlinear wave was extracted from the spectrogram of a signal receiving point. Tensile-driven plastic deformation and cumulative second harmonic generation of S_0_ mode were observed in the simulation. Simulated measurement of the acoustic nonlinearity increased monotonically with the level of tensile-driven plastic strain captured by MLPGEM, whereas achieving this state by other numerical methods is comparatively more difficult. This result indicates that the second harmonics of S_0_ mode can be employed to monitor and evaluate the material or structural early-stage damage induced by plasticity.

## 1. Introduction

An effective and reliable inspection technique for continuous monitoring and evaluation of early-stage nonlinearities in materials is necessary for engineering parts. Among all kinds of nondestructive methods studied for efficient damage detection and evaluation, the ultrasonic method is most useful and has been widely exploited for many decades [1,2,3,4]. Traditional ultrasonic inspection and monitoring methods are usually anchored on linear theory, and the characterization parameters are velocity attenuation, transmission, and reflection coefficients of the ultrasonic wave. However, conventional linear ultrasonic evaluation and monitoring methods are not sensitive to early stage micro-damage or micro-plastic deformation [4], and the smallest crack that current linear ultrasonic based approaches can monitor or evaluate is only approximately 1 mm [5]. This drawback limits their applications and does not allow early preventive actions [5,6,7].

When a high-intensity ultrasonic wave passes through a nonlinear medium, the waveform may be distorted [8]. It is well-known that a linear solid medium may become a nonlinear one gradually in the process of fatigue damage [9], radiation damage [10], hardening [11], and thermal aging [12] as the microstructural features are occupied by micro-voids, multi-poles, and micro-cracks [13], dislocation [14,15,16], persistent slip bands [17], and precipitation characteristics [18]. When a finite amplitude monochromatic sinusoidal ultrasonic wave interrogates a nonlinear solid, the initial waveform distorts and higher-order harmonic waves are generated. Overcoming the limitations of the linear ultrasonic method, the nonlinear ultrasonic technique measures higher harmonics generated by material intrinsic nonlinearity, and this technique has undergone rapid development in recent years. In most experiments or theoretical studies, only bulk waves are used, and a useful acoustic nonlinearity parameter presenting material nonlinearity is established by theoretical models [8,19,20]. However, bulk waves propagate with great energy loss and are not efficient in a large-scale inspection approach.

Guided waves, such as Lamb waves, combine large-scale monitoring or inspection ranges and the sensitivity of nonlinear parameters, which could be used for long-range monitoring or inspection to interrogate large shell- and plate-like structures. However, the monitoring or inspection techniques based on nonlinear Lamb waves might not be highly accurate because of their depressiveness and multiple modes. About the conditions to generate accumulative higher-order harmonic waves, corresponding theoretical studies may not be sufficiently clear and consistent because of the mathematical complexity of the problem [21,22,23,24,25,26,27]. To date, all theoretical studies agree that phase-velocity matching and non-zero power flux input from a primary mode to second harmonics are at least two necessary conditions for cumulative second harmonic generation. Mode pairs that satisfy these two conditions, such as S_1_–S_2_, S_2_–S_4_, and A_2_–S_4_ have been used in experimental studies. These nonlinear Lamb wave–based experiments have effectively characterized tensile plasticity driven damage [28], material nonlinearity [29], thermal fatigue [30], and fatigue damage [31], and have monitored the stress and load levels in strands [32]. However, using those higher-order mode as primary Lamb wave mode has some limitations in application [7]. First, in real applications, as the excitation signal is normally a tone burst with a finite bandwidth, strict phase velocity matching only exists at the center frequency of the whole bandwidth. Second, excited center frequency of the primary mode may not be exactly equal to the true phase-velocity-matching frequency. Third, mode pairs are isolated and quite limited in number. Fourth, exciting a single primary mode of the mode pairs as desired is extremely difficult in practice and some unwanted modes would be generated simultaneously, making the received signal quite difficult to be processed and interpreted. Finally, time-frequency analysis is needed to process the received complicated signals in which some unwanted Lamb modes overlap with the desired primary and secondary modes [7]. S_0_ mode Lamb wave could solve these limitations and this has been confirmed by many researchers. For instance, Wan et al. [7] used the cumulative effect of the second harmonic generated from S_0_ mode Lamb waves for the detection of microstructural changes [7]. Zuo et al. [33] calculated nonlinear harmonic of low frequency S_0_ wave for a 1.0 mm thick aluminum plate using the Murnaghan model, which is equivalent to the Landau–Lifshitz nonlinear hyper elastic constitutive model. Chillara and Lissenden [34] used the same Murnaghan model for an aluminum plate and found that the second harmonic can propagate independently to the primary mode and the group velocity matching is not necessary for higher harmonic generation by numerical study of S_0_ mode Lamb waves. By considering the effects of the second- and third-order elastic constants of isotropic media, Matsuda and Biwa [35] revealed that the frequency for which the second harmonic Lamb mode reaches maximum is close but not equal to the phase speed-matching frequency in a precise sense through theory and Finite-Difference Time-Domain method for S_0_ Lamb mode waves. Shan et al. [36] demonstrated that the nonlinear effects of the bonding layers in a typical PZT-actuated structural health monitoring system by using S_0_ mode Lamb waves. For micro-cracks, there are also some works using S_0_or A_0_Lamb waves [37,38,39,40,41,42,43,44]. However, there has been no work using the S_0_ mode Lamb wave to characterize tensile plasticity-driven damage.

It is well known that numerical exploration is attractive in ultrasonic nondestructive testing (NDT) to verify theoretical and experimental results and to find some new phenomena difficultly tackled by theory or experiment. The advantages of this method include ideal center frequency and single desired primary mode excitation [7], reduced cost, celerity, and simplicity. For instance, nonlinearities induced by buried micro-breathing cracks [32,37,38,39,40,41,42,43,44], material and geometry [45], and fatigue damage [46] are simulated through finite element method (FEM). The aforementioned simulations investigate on cumulative second-harmonic generation. In general, FEM, finite volume method, finite difference method, and boundary element method are suitable for simulating dynamic behaviors of continuum medium. However, special additional treatments, such as intensive remeshing to tackle mesh distortion caused by finite deformation and contact judgment, have to be considered in the calculation, where damage or fracture appears, simulating the entire failure procedure is difficult [47]. Under tensile loading, the plasticity-driven material damage and its evolution process is a problem that is difficult to simulate by commercial software such as ABAQUS or ANSYS. To ensure improved understanding of plasticity-driven cumulative second-harmonic generation, an alternative simulation method that could solve the problem is the discontinuous Meshless Local Petrov-Galerkin-Eshelby Method (MLPGEM), which is similar to the Discrete Element Method. It is very efficient for solving finite deformation nonlinear problems. For instance, it requires no explicit mesh in computation and therefore avoid mesh distortion difficulties and subsequent remeshing effort of traditional FEM in finite deformation analysis. Therefore, it should be a powerful tool to simulate the wave propagation in a largely deformed medium with the plasticity-driven material damage and its evolution process under tensile loading.

**Figure 1:**
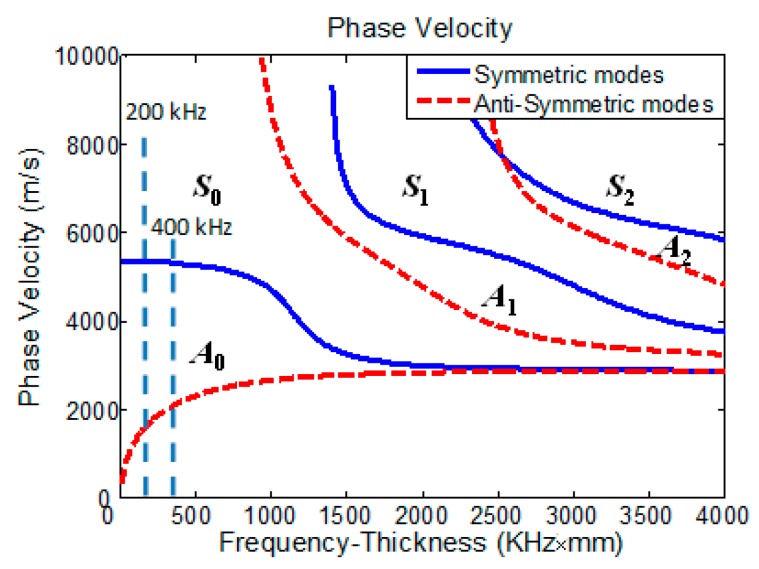
Dispersion curves of Lamb waves in an aluminum plate: phase velocity.

In this study, an MLPGEM module released by commercial software LS-DYNA is employed directly to deal with the above problem. The theory of MLPGEM and material plasticity is briefly explained as a foundation for simulating nonlinear Lamb waves. Unlike most previous works using those higher-order mode as primary Lamb wave mode [13,28,29,30,31,32], e.g., S_1_, S_2_, A_2_, etc., we focus on the case of S_0_ primary mode because of its remarkable merits [7] and investigate its second-harmonic generation. S_0_ Lamb wave with tone-burst cycles of 10 at the center frequency of 200 kHz has been used as a primary mode in the simulation. As shown in Figure 1, the condition of phase-velocity matching [21,22,23,24,25,31] can be satisfied approximately when considering S_0_ (400 kHz) as the second harmonic of this primary mode. The detailed descriptions of the numerical model as well as signal excitation and processing are presented. The results for the simulated material plasticity versus tensile strain and acoustic nonlinearity parameter corresponding to the different plasticity levels are provided and discussed. The results show that S_0_ Lamb wave and its corresponding second harmonic can be effectively employed to monitor and evaluate the plasticity driven early stage material damage.

## 2. Material Nonlinearity and Its Measurement

Theoretical models for longitudinal waves in one-dimensional medium [46] and Lamb waves in isotropic, homogeneous, and nonlinear elastic infinite plate [22,27] show that the acoustic nonlinearity parameter of a material, i.e., *β* can be defined as,

(1) $$\beta =\frac{8}{xk^{2}}(\frac{A_{2}}{A_{1}^{2}})$$

where *k* is wavenumber, *x* is wave propagation distance, *A*_1_ is magnitude of the primary wave mode at *ω*, and *A*_2_ is second harmonic wave mode at 2*ω*. The amplitude ratio $A_{2}/A_{1}^{2}$ is a measure of the nonlinearity parameter *β* and is widely used in experiments and simulations [38,45,48,49,50,51]. A monotonic increase between *β* and the accumulated plasticity of some metals was confirmed for bulk waves [17,52] and Lamb waves with S_1_–S_2_ pair [28]. Therefore, the tensile-plasticity-driven material damage might be characterized by directly measuring $A_{2}/A_{1}^{2}$.

## 3. Theory and Model of MLPGEM 

### 3.1. Theory

**Figure 2:**
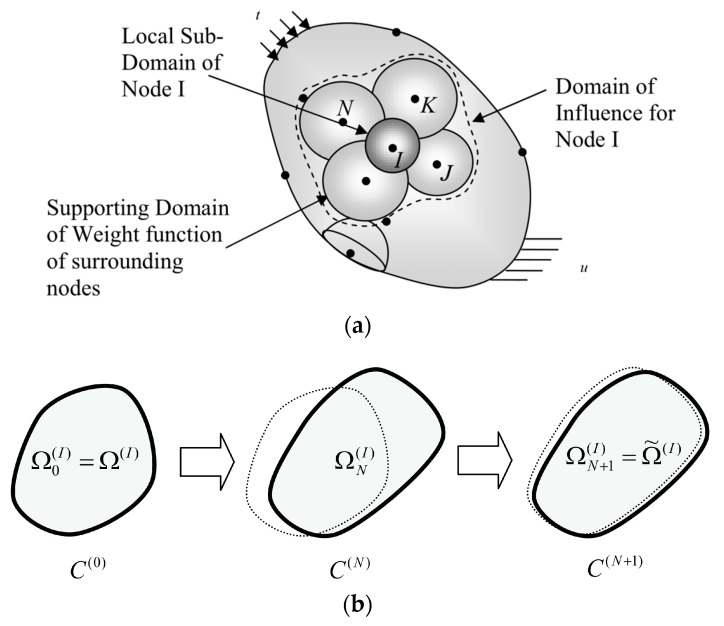
Description of MLPGEM: (**a**) trial and test domain; (**b**) three configurations during a finite deformation.

MLPGEM combines the Meshless Local Petrov-Galerkin (MLPG) Methods of Atluri and the energy conservation laws of Noether and Eshelby [53]. MLPG is a truly meshless method that involves both non-element interpolation and non-element integration [54]. MLPG may alleviate problems such as element distortion, locking, remeshing during large deformation process. With the help of the “energy-momentum tensor” given by Eshelby, Han and Atluri solved the singular integrals appearing in the local boundary integral equation and proposed a numerical method called MLPGEM [53]. This method is simply described as follows: a body is assembled by spheres (in 3-D or circles in 2-D) centered at each meshless node. The moving least squares (MLS) approximation over a number of nodes within the domain of influence is used to construct the trial functions. Trial functions is based on the fictitious nodal value of the undeformed configuration. The test functions for configurational changes of the deformed configuration are also based on meshless nodes. While the local sub-domain is defined as the support of the test function on which the integration is carried out, the domain of influence is defined as a region where the weight function of the node inside of it does not vanish in the local sub-domain of the current node. In other words, the domain of influence contains all the nodes that have non-zero coupling with the current nodal values in the stiffness matrix. In Figure 2a, the sphere surrounding node I represents the local sub-domain where the integration is carried out. The spheres surrounding node J, K, L, M… represent the supporting domain of weight functions of nodes, whose weight functions do not vanish in the local sub-domain. The volume surrounded by dashed curve represents the domain of influence of node I. In general, the size of the domain of influence has to be large enough in order to ensure continuity. Piecewise-linear predictor solutions are generated based on the local weak-forms of the Noether/Eshelby energy conservation laws for the Lagrangian unsymmetric Eshelby stress tensor in the undeformed configuration. Corrector solutions are generated based on Newton-Raphson (or Jacobian-inversion-free iterations) using the local weak-forms of the Noether/Eshelby energy conservation laws in the current configuration, for a newly introduced Eulerean symmetric stress tensor (which is the counter part of the Lagrangian unsymmetric Eshelby stress tensor) in the current configuration.

We consider the finite deformation of a solid, wherein a material particle initially at **X**, moves to a location **x**. We use a fixed Cartesian coordinate system with base vectors $e_{i}$, such that,

(2) $$X=X_{I}e_{i}$$

(3) $$x=x_{i}e_{i}$$

The displacement of the material particle is,

(4) $$u(X)=x(X)-x\;\mathrm{or}\;u_{i}=(x_{i}-X_{I})e_{i}$$

The deformation gradient tensor is defined as,

(5) $$F\;[F_{iJ}=\frac{\partial x_{i}}{\partial X_{J}}\equiv x_{i,J}=u_{i,J}+\delta_{iJ}]$$

There are infinitely many possible definitions of a stress-tensor in a finitely deformed solid. Among them, the more commonly used ones are: the Cauchy stress tensor *σ*; the first Piola-Kirchhoff stress tensor **P**; and the second Piola-Kirchhoff stress **S**, which are related to each other, thus,

(6) $$P=JF^{-1}\sigma =S\cdot F^{t};S=JF^{-1}\cdot \sigma \cdot F^{-t}=P\cdot F^{-t}\;[J=\left\|F\right\|]$$

Considering a general anisotropic solid, with the strain energy per unit initial volume being denoted as *W*, the constitutive relation for **P** may be written as,

(7) $$P=\frac{\partial W}{\partial F^{t}}$$

The equations of Linear Momentum Balance (LMB) and Angular Momentum Balance (AMB) can be written equivalently in terms of *σ*, **P**, and **S**, as,

(8) $$\begin{array}{l} \sigma_{ij}{}_{,i}+\rho f_{j}=0;\;(LMB);\;\sigma =\sigma^{t}\;(AMB)\\ P_{Ij}{}_{,I}+\rho_{0}f_{j}=0;\;(LMB);\;F\cdot P=P^{t}\cdot F^{t}\;(AMB)\\ {\left(S_{IK}F_{jK}\right)}_{,I}+\rho_{0}f_{j}=0;\;(LMB);\;S=S^{t}\;(AMB) \end{array}$$

The Eshelby stress tensor is defined, for finite deformations, as,

(9) $$T=WI-P\cdot F=WI-S\cdot F^{t}\cdot F=WI-S\cdot C$$

The strong form balance laws for the Eshelby stress tensor **T** is given as,

(10) $$T_{IJ,I}+\rho_{0}b_{J}=-(P_{Ik,I}+\rho_{0}f_{k})F_{kJ}=0,(LMB),C\cdot T=T^{t}\cdot C,(AMB)$$

where, by definition,

(11) $$b_{J}\equiv \frac{1}{\rho_{0}}{W_{,J}|}_{\exp.}+f_{k}F_{kJ}$$

We refer the solution variables (displacements, deformation gradient, and stresses) in the state $C^{\left(N+1\right)}$to the configuration of the body in the immediately preceding state, denoted as $C^{\left(N\right)}$, which is presumed to be known, including the variables in $C^{\left(N\right)}$, such as **u**, **F**, *σ*, and **T**, with the initial configuration $C^{\left(0\right)}$being the reference configuration, as shown in Figure 2b. Let $\tilde{v}(X)$ be the configurational changes of the initial configuration which are the trial functions in the present MLPGEM, and let the corresponding changes to the displacements in $C^{\left(N\right)}$ as given in equation $\Delta u=F\cdot \tilde{v}(X)$. One may have the spatial tangential material stiffness for $\Delta S_{IJ}$ in the configuration $C^{\left(N\right)}$, derived as,

(12) $$\Delta S_{IJ}=\frac{1}{2}\frac{\partial^{2}W}{\partial E_{IJ}\partial E_{MN}}F_{kM}F_{lN}[F_{kK}F_{Ll}^{-1}+F_{Lk}^{-1}F_{lK}]\Delta L_{KL}^{*}\equiv C_{IJKL}^{*}\Delta K_{KL}^{*}$$

The local weak-forms of the energy conservation laws can be derived as,

(13a) $$\begin{array}{l} \left\{\int_{\partial \Omega_{N}^{\left(I\right)}}n_{i}c_{ijKL}^{\tan\mathrm{gent}}\Delta K_{KL}^{*}\delta x_{j}dS-\int_{\Omega_{N}^{\left(I\right)}}c_{ijKL}^{\tan\mathrm{gent}}\Delta K_{KL}^{*}\delta x_{j,i}d\Omega \right\}+\left\{\int_{\partial \Omega_{N}^{\left(I\right)}}\rho_{N}\Delta f_{j}\delta x_{j}d\Omega \right\}\\ +\left\{\int_{\partial \Omega_{N}^{\left(I\right)}}n_{i}\sigma_{ij}^{\left(N\right)}\delta x_{j}dS-\int_{\Omega_{N}^{\left(I\right)}}\sigma_{ij}^{\left(N\right)}\delta x_{j,i}d\Omega +\int_{\Omega_{N}^{\left(I\right)}}\rho_{N}f_{j}^{N}\delta x_{j}d\Omega \right\}=0,\\ c_{ijKL}^{*}\equiv \frac{1}{J^{\left(N\right)}}{\left(F_{0}^{N}\right)}_{iM}\cdot C_{MNKL}^{*}\;\cdot {\left(F_{0}^{N}\right)}_{jN}\;\mathrm{and}\;c_{ijKL}^{\tan\mathrm{gent}}=c_{ijKL}^{*}+\sigma_{IM}^{\left(N\right)}F_{jK}F_{KL}^{-1}\\ \mathrm{and}\;\sigma^{\left(N\right)}=\frac{1}{J^{\left(N\right)}}(F_{0}^{N})\cdot (S_{0}^{N})\cdot {\left(F_{0}^{N}\right)}^{t}. \end{array}$$

With a constant test function δx over each local sub-domain, the domain integrals vanish and the local weak-forms of the energy conservation laws may be further simplified as,

(13b) $$\begin{array}{l} \left\{\int_{\Omega_{N}^{\left(IJ\right)}}\frac{1}{2}[(c_{ijKL}^{\tan\mathrm{gent}}\Delta L_{KL}^{*}{)}^{\left(J\right)}+({\left(c_{ijKL}^{\tan\mathrm{gent}}\Delta L_{KL}^{*}\right)}^{\left(I\right)}](\delta x_{j}^{J}-\delta x_{j}^{I})dS\right\}=\left\{\begin{array}{l} \int_{\partial \Omega_{N}^{\left(I\right)}}\rho_{N}\Delta f_{j}\\ \delta x_{j}d\Omega \end{array}\right\}\\ +\left\{\int_{\Omega_{N}^{\left(I\right)}}\rho_{N}f_{j}^{N}\delta x_{j}d\Omega -\int_{\partial \Omega_{N}^{\left(IJ\right)}}\frac{1}{2}[{\left(\sigma_{ij}^{\left(N\right)}\right)}^{\left(J\right)}+{\left(\sigma_{ij}^{\left(N\right)}\right)}^{\left(I\right)}](\delta x_{j}^{J}-\delta x_{j}^{I})dS\right\} \end{array}$$

Once the trial functions $\tilde{v}(X)$ (configurational changes in the initial configuration) are solved through Equation (13), corresponding to either the unbalanced force or to the incremental loading, the corresponding displacements can be obtained through $\Delta u=F\cdot \tilde{v}(X)$. It is clear that Equation (13) can be applied in both Total Lagrangian formulation or an Updated Lagrangian formulation by setting the displacements **u**(**X**) of the preceding solution accordingly. In Equation (13b), $c_{ijKL}^{\tan\mathrm{gent}}$ is the quantity to reflect the current material properties, e.g., elastic or plastic behavior at different loading stages in the analysis, which can be understood as the nonlinear stiffness connecting nodes or spheres in Figure 2a. One may see further details in the references [53,54,55].

### 3.2. Model

**Figure 3:**
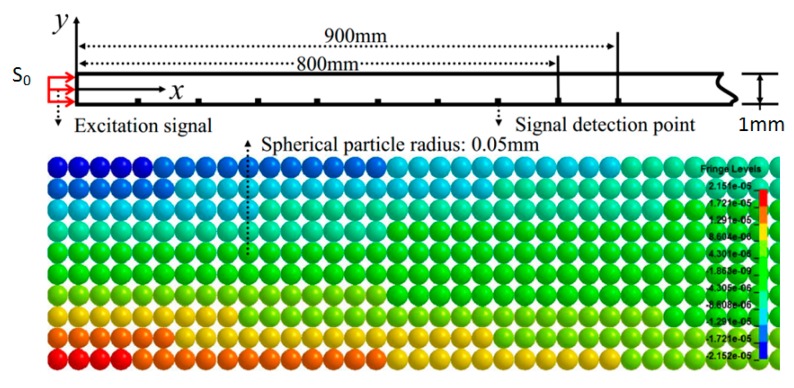
Meshless Local Petrov-Galerkin-Eshelby Method (MLPGEM) model.

Figure 3 shows the MLPGEM model (Livermore Software Technology CORP., California, CA, USA). The Lamb waves of S_0_ mode propagating in an aluminum plate is symmetrical in the *y*-axis direction. For simplicity, the plate with thickness 1 mm could be modeled in two dimensions by assemblies of small spheres. The model should be long enough (2.0 m) to ensure that the received signals are not affected by boundary reflections.

A loading process can be described as: a tensile load is slowly applied on the plate which makes the plate be deformed into a state needed, and the deformed plate is hold to keep this deformation. Lamb waves of S_0_ mode in terms of a designed function are then generated with the line source signal excitation acting on the left side of the present plate. The loading function of the above process can be described as,

(14) $$x(t)=\{\begin{array}{c} \frac{l}{2}(\cos(\frac{2\pi }{T}t)-1)\\ -l\\ \frac{A}{2}\sin(2\pi ft)(1-\cos(2\pi \frac{f}{N}t))-l\\ -l \end{array}\begin{array}{c} t\in [0,\frac{T}{2})\\ t\in [\frac{T}{2},t_{1})\\ t\in [t_{1},t_{2})\\ t\in [t_{2},t_{e}] \end{array}$$

where *l* is the tensile length in mm, *T* is the period of tensile function in ms, *f* is the central frequency in kHz, *N* (=10) is the number of sinusoidal cycles in a wave pulse, $\frac{T}{2}$ is the end time of the tensile process, *A* (=0.01 mm) is the amplitude of tone burst, *t*_1_ is the end time of the holding process, *t*_2_ is the end time of the loading (Hamming windowed tone-burst consisting of 10 cycles at the center frequency 200 kHz), and *t_e_* is the end time of the simulation.

**Figure 4:**
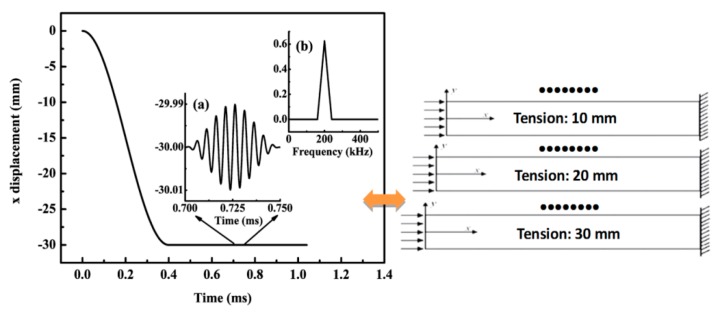
Actuating function of excitation signal with stretching length *l* = 30 mm and *T* = 0.8 ms: (**a**) time domain and (**b**) frequency domain.

When *l* is equal to 30 mm—for example, the loading function is shown in Figure 4—the plate is stretched by 30 mm with a strain of 0.015. Seven different *ls* (i.e., 0 mm, 5 mm, 10 mm, 15 mm, 20 mm, 25 mm, and 30 mm) were loaded in our model. A large *l* needs a large *T* and *t*_1_. In our simulation, nine signal receiving points are considered with a distance from 100 mm to 900 mm with an interval of 100 mm from the left of the model, where the excitation signal actuated, as illustrated in Figure 3.

**Figure 5:**
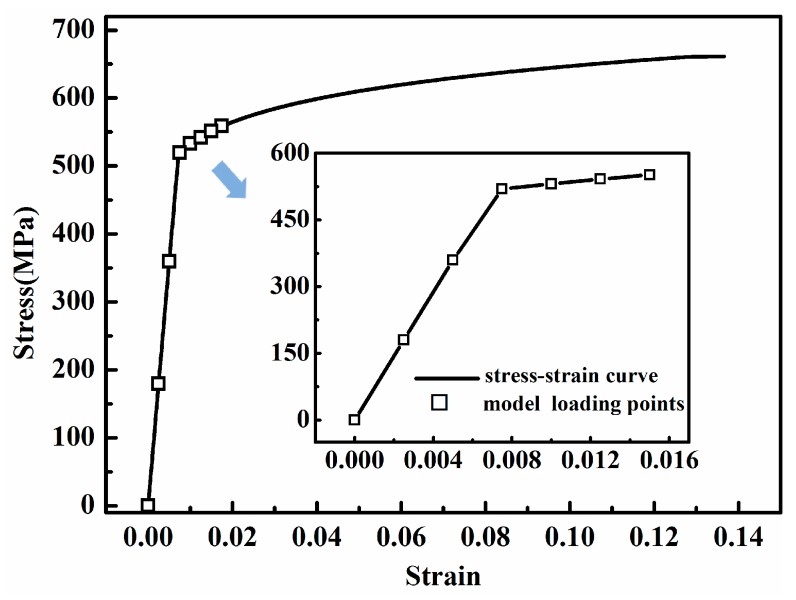
Stress–strain curve of Al 7075-T651 plate with maximum strains loaded for the seven models.

The MLPGEM module of LS-DYNA with the Newton-Raphson iterations method is used in this study. For fundamental signal 200 kHz and higher harmonics 400 kHz, a mesh size of 0.1 mm (particle radius 0.05 mm) and a time step of 0.02 μs are suitable and enough to ensure accuracy [56,57]. For undamaged Al 7075-T651 plate, piecewise linear plasticity with failure was chosen as the material model, and the stress–strain constitutive relationship is shown in Figure 5. In the present work, the plasticity of aluminum 7075-T651 was represented by a nonlinear stress-strain curve. The practical experimental tensile stress-strain curve was used. We explain our modelling as follows: the nonlinear constitutive law is a two-stage one, i.e., the linear elastic stage and nonlinear plastic stage after yielding as shown in Figure 5. The Mises yield surface is used to judge isotropic yielding. It is defined by giving the value of the uniaxial yield stress as a function of uniaxial equivalent plastic strain. Below the yielding strength, the material behavior was modeled in a linear way as shown in Figure 5. After the yielding, the material plastic behavior was modeled in a piecewise linear pattern using some discrete points as shown in Figure 5. This plasticity should be considered to be caused by dislocations in the material, which is different to hyper-elasticity. Therefore, we employ a classical phenomenological plastic model here. Our method, unlike the theoretical way to deal with material nonlinearity, does not use higher-order expansion of the constitutive relation between the applied stress and resulting strain.

In fact, a material table was defined relating the different strain-levels to the stress levels. The seven representative tensile stresses with the corresponding maximum strains at different levels represented by a symbol of square are also plotted in Figure 5. The first four points simulate the elastic zone and they form a straight line. The nonlinear stress-strain zone is approximated using the piecewise linear model with more discrete points. The material properties are shown in Table 1.

## 4. Simulation Results and Discussion

### 4.1. Effectiveness Validation of MLPGEM

**Figure 6:**
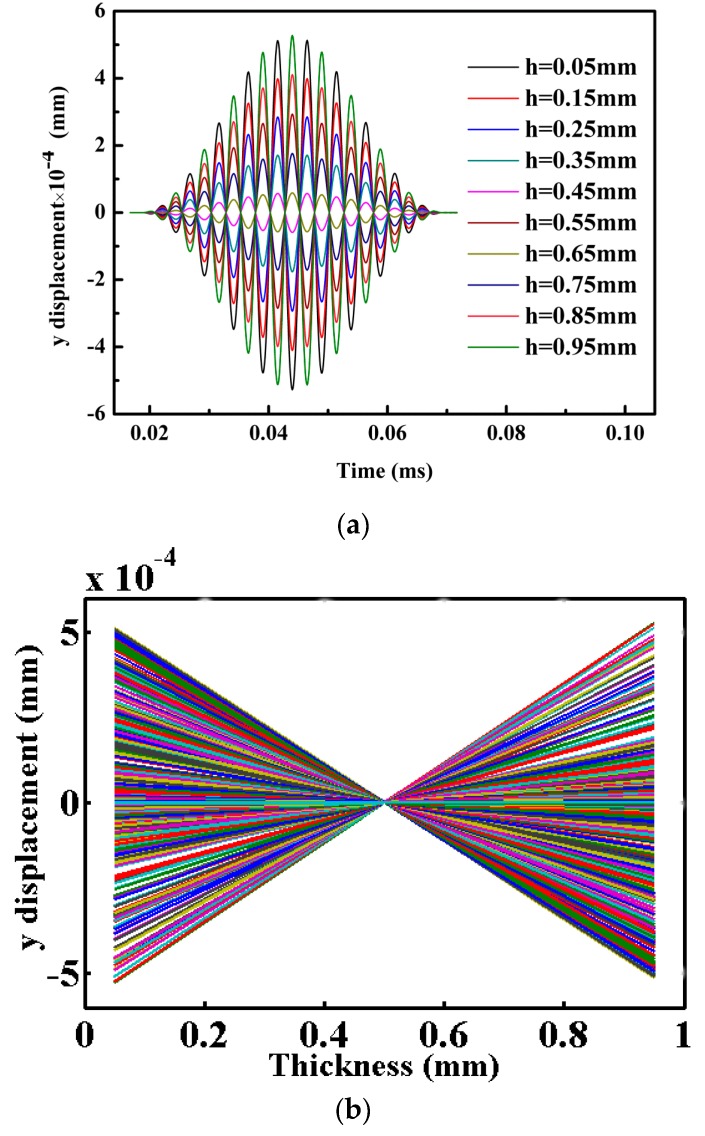
(**a**) Displacement in *y*-axis direction at different locations through-thickness direction with the same *x* coordinate (=100 mm); (**b**) S_0_ mode at 200 kHz—through thickness profiles for *y* displacement at *x* coordinate (=100 mm) for various times *t* = 0.02–0.07 ms.

**Figure 7:**
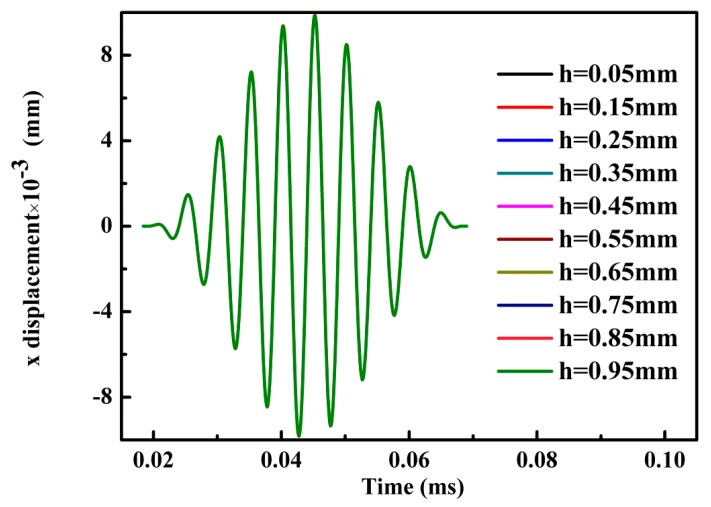
Displacement in *x*-axis direction at different locations through-thickness direction with the same *x* coordinate (=100 mm).

For an unloaded plate, Figure 6a,b and Figure 7 indicate that the through thickness profile for displacement in *y*-axis direction is antisymmetric and for displacement in *x*-axis direction is symmetric. Note that *h* denotes the distance from the bottom surface of the plate and all curves in Figure 7 are overlapped. Thus, a pure symmetric mode Lamb wave (S_0_) with center frequency of 200 kHz is excited in our MLPGEM model. To verify the validity of our model further, group velocity verification for Lamb waves of symmetric and antisymmetric modes at center frequency of 100 kHz and 200 kHz are tested. Note that for A_0_ mode, the shear stress was applied at the left end of the plate in Figure 2. The velocity results shown in Table 2 agree with the theoretical results within the allowable error range. Based on the preceding research, the validity of our MLPGEM model is verified.

### 4.2. Simulation Results and Discussion

**Figure 8:**
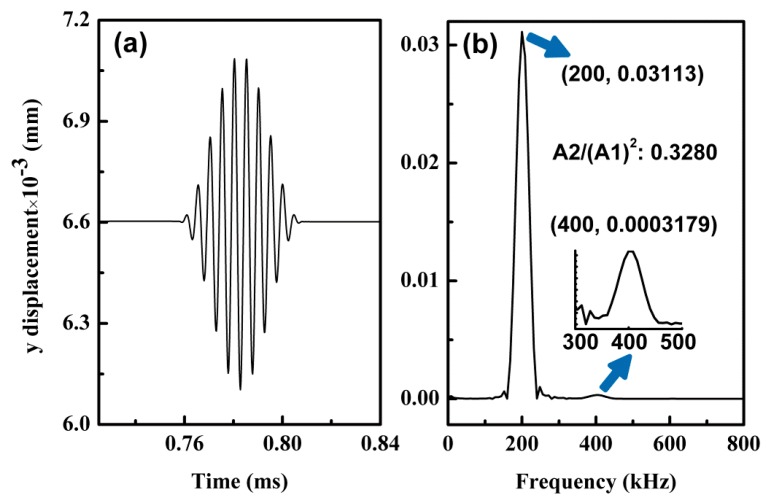
Signal received at propagation distance of 300 mm for S_0_ at 200 kHz with maximum strain 0.015 (tension 30 mm): (**a**) time domain and (**b**) frequency domain; amplitude ratio is 0.3280.

**Figure 9:**
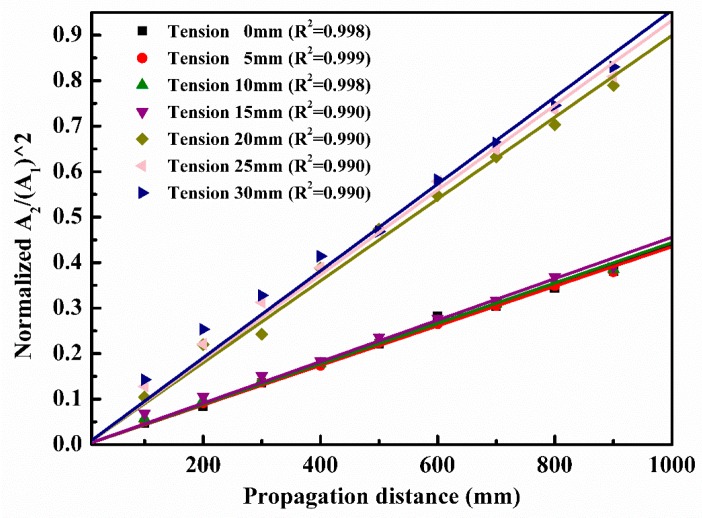
Amplitude ratio $A_{2}/A_{1}^{2}$, a measure of the nonlinearity parameter *β*, plotted as a function of propagation distance for the seven models (linearly fitted lines passing through the origin).

Figure 8a (time domain) and Figure 8b (frequency domain) show the received signal when Lamb waves propagate through 300 mm. As indicated in Figure 8b, second harmonics are generated at an amplitude ratio of 0.3280, denoted by $A_{2}/A_{1}^{2}$. For each *l* (0 mm, 5 mm, 10 mm, 15 mm, 20 mm, 25 mm, and 30 mm) in the actuating function described in Equation (14), we computed the amplitude ratios of the received signals from the nine signal receiving points, and then we plotted the results in Figure 9 to compare and analyze for convenience. In the signal processing using Fast Fourier Transformation (FFT), the window length of a rectangular function was adjusted continuously (but valid signals were not cut off) until the center frequency of the primary fundamental wave was 200 kHz. We could then obtain comparatively more accurate and consistent data.

**Figure 10:**
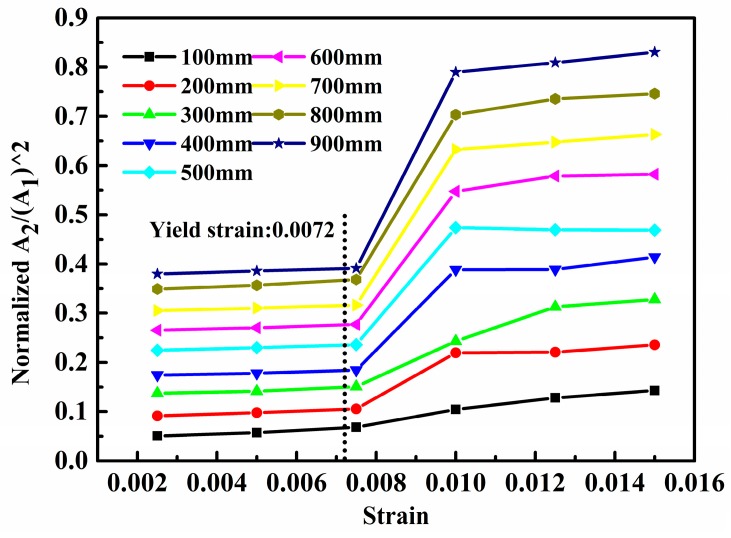
Normalized acoustic nonlinearity versus strain with the model.

Figure 9 shows the amplitude ratio, i.e., $A_{2}/A_{1}^{2}$, which is a linear function of propagation distance. The lines can be categorized into two groups. The first group includes tensile strains 0.01 (*l* = 20 mm), 0.0125 (*l* = 25 mm), and 0.015 (*l* = 30 mm, note that the yield strain is 0.0072) with larger slopes. The second group consists of tensile strains 0, 0.0025 (*l* = 5 mm), 0.005 (*l* = 10 mm), and 0.0075 (*l* = 15 mm) with smaller slopes. The slopes of the lines in each group are too close to distinguish. In every group at the same propagation distance, the amplitude ratio is slightly larger when the stretching deformation is larger. Thus, the greater the tension, i.e., plasticity extent, is, the larger is the amplitude ratio (acoustic nonlinearity). When the tensile strain exceeds the yield stain to an extent, a remarkable increase of the amplitude ratio occurs suddenly as concluded more clearly in Figure 10.

Figure 10 shows a plot of $A_{2}/A_{1}^{2}$ versus the strain of the Lamb wave. The signal receiving points range from 100 mm to 900 mm with an interval of 100 mm. Note that when we used different amplitudes *A* of excitation signals in Equation (2) (e.g., 0.001 mm and 0.1 mm) or different frequencies (e.g., 100 kHz and 300 kHz), the change tendencies of $A_{2}/A_{1}^{2}$ in Figure 9 and Figure 10 were similar. Figure 10 shows that the amplitude ratio at different propagation distances exhibit the similar variation pattern. At the yield strain, i.e., 0.0072, $A_{2}/A_{1}^{2}$ increases remarkably. Compared with those inelastic stage, $A_{2}/A_{1}^{2}$ in plasticity deformation have an approximate 200% increase at around 0.01 tensile strain. For further higher plastic strain levels, both Figure 9 and Figure 10 show that the amplitude ratio increases only slightly. This feature of increasing trend in $A_{2}/A_{1}^{2}$ of is similar to what the theoretical models [17,52,58] for bulk waves predict essentially and has been verified by experiments when using those higher-order modes of Lamb waves as primary mode, e.g., S_1_ or S_2_ [28]. The amplitude ratio increases remarkably at the happening of plasticity and this increase becomes much weaker with subsequent accumulated plasticity. Thus, Lamb waves of S_0_ mode can be used to evaluate plasticity driven material damage, or at least is capable of characterizing the occurring of plasticity.

In the simulation process of tension, geometrical discontinuous defects (such as cracks, voids, dislocations, and impurity) were not modelled. However, $A_{2}/A_{1}^{2}$ caused by these defects is completely reflected in the stress–strain curve in the present simulations, i.e., material softening. 

Therefore, the present model is reasonable for simulating material plasticity. Moreover, the above results imply that $A_{2}/A_{1}^{2}$ based on S_0_ mode can be used to characterize plasticity-driven material damage, or at least, to monitor the occurring of plasticity. After entering into the plasticity, with the further increase of tensile strain, there are only slight increases in $A_{2}/A_{1}^{2}$, although this $A_{2}/A_{1}^{2}$ is around two times higher thanthat of material in elastic deformation. Whether $A_{2}/A_{1}^{2}$ can be used to accurately characterize the plasticity extent or not still needs more extensive explorations.

The above simulation results are also verified by some experiments reported by others indirectly. For example, C. Pruell et al. [28] develops an experimental method for evaluating damage due to plastic deformation in a metal plate using nonlinear-guided waves. The material nonlinearities of aluminum specimens loaded to produce different levels of plastic strain are measured with Lamb waves. A pair of wedge transducers is used to generate and detect the fundamental (S_1_) and second harmonic (S_2_) Lamb waves. The weak amplitude of the second harmonic wave is extracted from the spectrogram of a received signal. The measured acoustic nonlinearity increases monotonically with the level of plastic strain, which is similar to our simulations. After the plasticity stage, with the increase of tensile strain, there is almost no obvious increase in the normalized acoustic nonlinearity (NAN), although this NAN is around two times higher than that of material in elastic deformation. Hong et al. [46] claim the acoustic nonlinearity parameter grows with the propagation distance, which corresponds with the tendency of results showed in Figure 9. Jhang et al. [59,60] performed the acoustic nonlinearity testing on samples with different stresses. The results show that the acoustic nonlinearity increase with the tensile load, which is also consistent with Figure 10. To summarize, the simulation result is reasonable.

## 5. Conclusions

This investigation demonstrates that MLPGEM can be used to simulate and evaluate plasticity driven material damage based on nonlinear S_0_ mode Lamb waves in metallic plates. Simulated results are in accordance with previous theoretical analysis and experimental research for bulk waves or higher-order modes of Lamb waves as primary mode. The results also show that nonlinear Lamb waves based on S_0_ mode can be used to assess plasticity driven material damage, or at least monitor the occurrence of plasticity. Further research could develop MLPGEM models with micro-cracks (micro-breathing cracks), dislocations, thermal fatigue, voids, and impurity defects.

## Figures and Tables

**Table 1:** Material properties of Al 7075-T651.

Material	$\rho ({g/\mathrm{mm}}^{3})$	E (MPa)	Poisson’s Ratio	Yield Stress (MPa)
Al 7075-T651	2.7957 × 10^−3^	7.1705 × 10^4^	0.33	517.84

**Table 2:** Group velocity verification for symmetric (S_0_) and antisymmetric (A_0_) mode Lamb waves at center frequencies of 100 kHz and 200 kHz.

Mode Type and Frequency (kHz)	Theoretical Velocity (m/s)	Simulation Velocity (m/s)	Error (%)
S_0_ (100 kHz)	5444	5263	3.32
S_0_ (200 kHz)	5437	5235	3.72
A_0_ (100 kHz)	1748	1707	2.35
A_0_ (200 kHz)	2283	2252	1.36
